# Research hotspots and development trends in volume management of peritoneal dialysis patients: a bibliometrics and visual analysis up to 2022

**DOI:** 10.1007/s11255-023-03869-7

**Published:** 2023-11-22

**Authors:** Tingting Liu, Dan Zhao, Jiaying Huang, Aiping Gu, Qian Liu, Wei Fang, Leyi Gu, Haifen Zhang

**Affiliations:** 1 Department of Nursing, Ren Ji Hospital, Shanghai Jiao Tong University School of Medicine, Shanghai, People’s Republic of China; 2 Department of Nephrology, Ren Ji Hosptial, Shanghai Jiao Tong University School of Medicine, Shanghai, People’s Republic of China

**Keywords:** Peritoneal dialysis, Volume management, Visualization, Research hotspots, Bibliometric analysis

## Abstract

**Objectives:**

Among different renal replacement therapies (RRTs), peritoneal dialysis (PD) is a family based treatment method with multiple advantages, which allowing patients to maintain autonomy, avoiding frequent hospital visits, and preventing the spread of the disease virus. To visually analyze the literatures related to volume management of PD patients through bibliometric methods, to explore research hotspots and development trends in this field.

**Methods:**

The relevant literatures of PD patient volume management in the Web of Science core collection database were retrieved with the terms of peritoneal dialysis, volume management, capacity management, fluid status, and volume overload. The retrieval time was from the establishment of the database to October 2022. CiteSpace 6.1.R3 software was used to visually analyze Country, Institution, Author, Keyword, and draw keyword clusters and keyword emergence maps.

**Results:**

A total of 788 articles were included in the analysis, and the annual number of papers was on the rise, with the American, China, and Brirain in the top three, and Peking University and University College London in the top. Keywords cluster analysis showed 11 clusters. In the keyword emergence analysis, the keywords with higher emergence intensity rank are continuous cyclic peritoneal dialysis, ambulatory peritoneal dialysis, and icodextrin. The current research hotspots and trends are in the evaluation of peritoneal dialysis patients’ volume status, the selection and adjustment of dialysis prescriptions, and adverse health outcomes.

**Conclusion:**

The research on peritoneal dialysis volume management in China started late, but it has developed rapidly, and has a firm grasp of current research hotspots. However, there is less cooperation with other countries, so international exchanges and cooperation should be strengthened. At present, the volume assessment methods and dialysis modes are still the research hotspots, paying more attention to the adverse health outcomes of patients.

## Introduction

Peritoneal dialysis (PD) is one of the renal replacement therapies (RRT) for end-stage kidney disease patients. It is a family-based and cost-effective treatment that has a relatively small impact on patients’ daily lives and has its unique advantages during the pandemic [1]. With the continuous development of PD technology, the survival rate and technical survival rate of PD patients are constantly improving, and cardiovascular disease is the primary cause of death in PD patients. Long-term PD patients are prone to abnormal volume balance, manifested as overload or insufficiency. Volume imbalance is closely related to adverse prognosis such as cardiovascular disease in PD patients [2]. Research shows that the incidence rate of left heart failure in PD patients with volume overload is higher than that in patients with normal volume [3], and the risk of diabetes, hypertension, and hypoproteinemia is also increased. Volume management is one of the treatment goals for PD patients [4], and conducting research on volume management for PD patients is of great significance. To systematically mine the main content, central tendency, and characteristics of PD patient volume management research, this study uses the method of knowledge mapping to conduct visual analysis of related research. Based on structured, semi-structured, or unstructured data sources, knowledge graphs can extract data and visualize the relationships between data in the form of graphs. It can be recognized and understood by both humans and machines, helping researchers analyze problems from the relationships between data. CiteSpace can use visual knowledge graphs to showcase the knowledge foundation, research frontiers, evolution process, and development trends within the discipline [5]. This study uses CiteSpace 6.1.R3 software to sort out relevant literatures in this field, and from the perspective of visual analysis, summarizes the current research hotspots and development trends in PD patient volume management in the form of knowledge graphs, aiming to provide reference for future research.

## Methods

This study is based on the Web of Science core collection database [6], using TS = (periodic analysis) AND (TS = (volume management) OR TS = (capacity management) OR TS = (fluid status) OR TS = (volume overload)) as the search method for topic retrieval. The search was conducted until October 2022, and the language limit was English. A total of 1080 articles were retrieved. Exclude edited materials, letters, conference papers, notes, conference record files, and republished literatures. After reading the title and abstract, exclude literatures that clearly does not match the topic. Utilize the Remove Duplicates function in CiteSpace software, and ultimately include 788 relevant literatures.

## Statistical analysis

Import the literatures into the software, set the time span (Time Slicing) according to the publication time of the literatures, set the Time Slice (Years Per Slice) to 1 year, set the node threshold to Top *N* = 50, set the clipping method to Pathfinder Running sliced networks, and select default values for other parameters. Visualization analysis is conducted using literatures such as Country, Institution, Author, and Keyword as nodes. The national cooperation network and keyword co-occurrence graph are presented in the form of a ring chart. The larger the ring diameter, the higher the frequency. The node circle layer represents the ring, and the color range from light gray to warm red indicates the year from far to near. The betweenness centrality is a concept based on CiteSpace's own scientific metrology theory. It is an indicator reflecting the importance of nodes in the network. The nodes whose intermediary centrality exceeds 0.1 are called key nodes. Clustering modularization value (*Q* value) > 0.3 indicates significant clustering structure, clustering average contour value (*S* value) > 0.5 indicates reasonable clustering, and *S* > 0.7 indicates reliable clustering structure.

## Results

### Annual publication trends

**Fig. 1:**
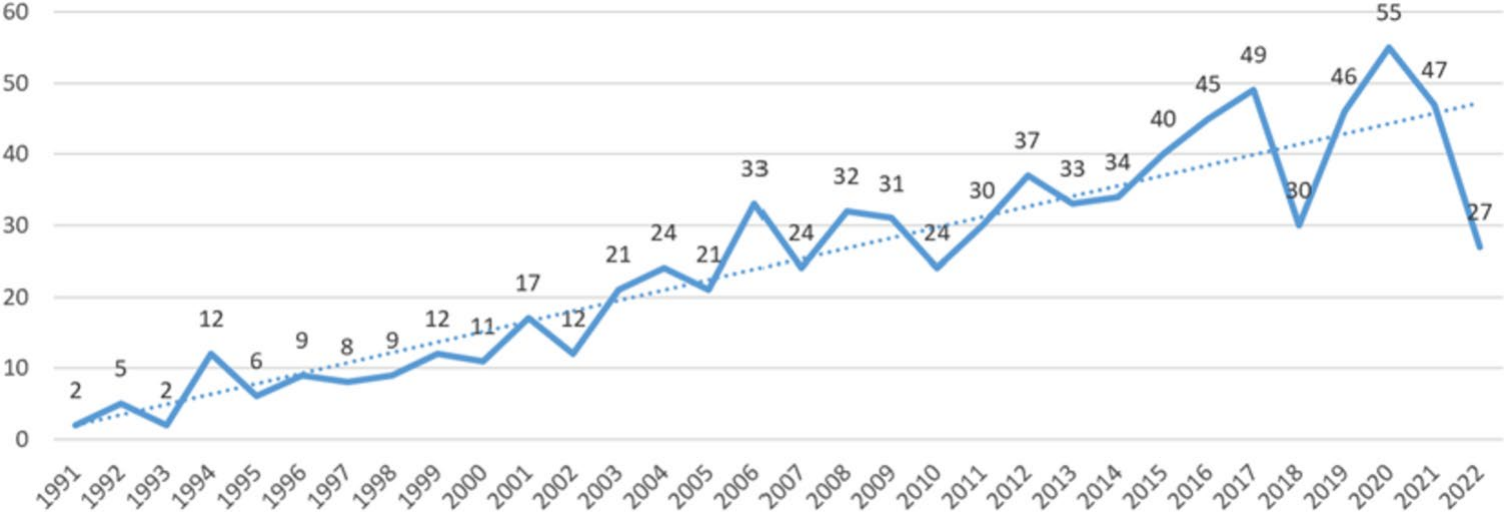
Annual publication volume of PD volume management

The temporal distribution characteristics of relevant literatures in the field can directly reflect the development speed and research hotspots of the field. In 1991, relevant literatures in the field of PD patient volume management were first searched, with an annual publication volume of two articles. By 2022, there was a slight fluctuation in the publication volume, but the overall trend was on the rise. Among them, the literatures’ volume significantly decreased in 2018, with an annual publication volume of 30 articles. In 2020, it reached its peak at 55 articles (Fig. 1).

### Country distribution

**Fig. 2:**
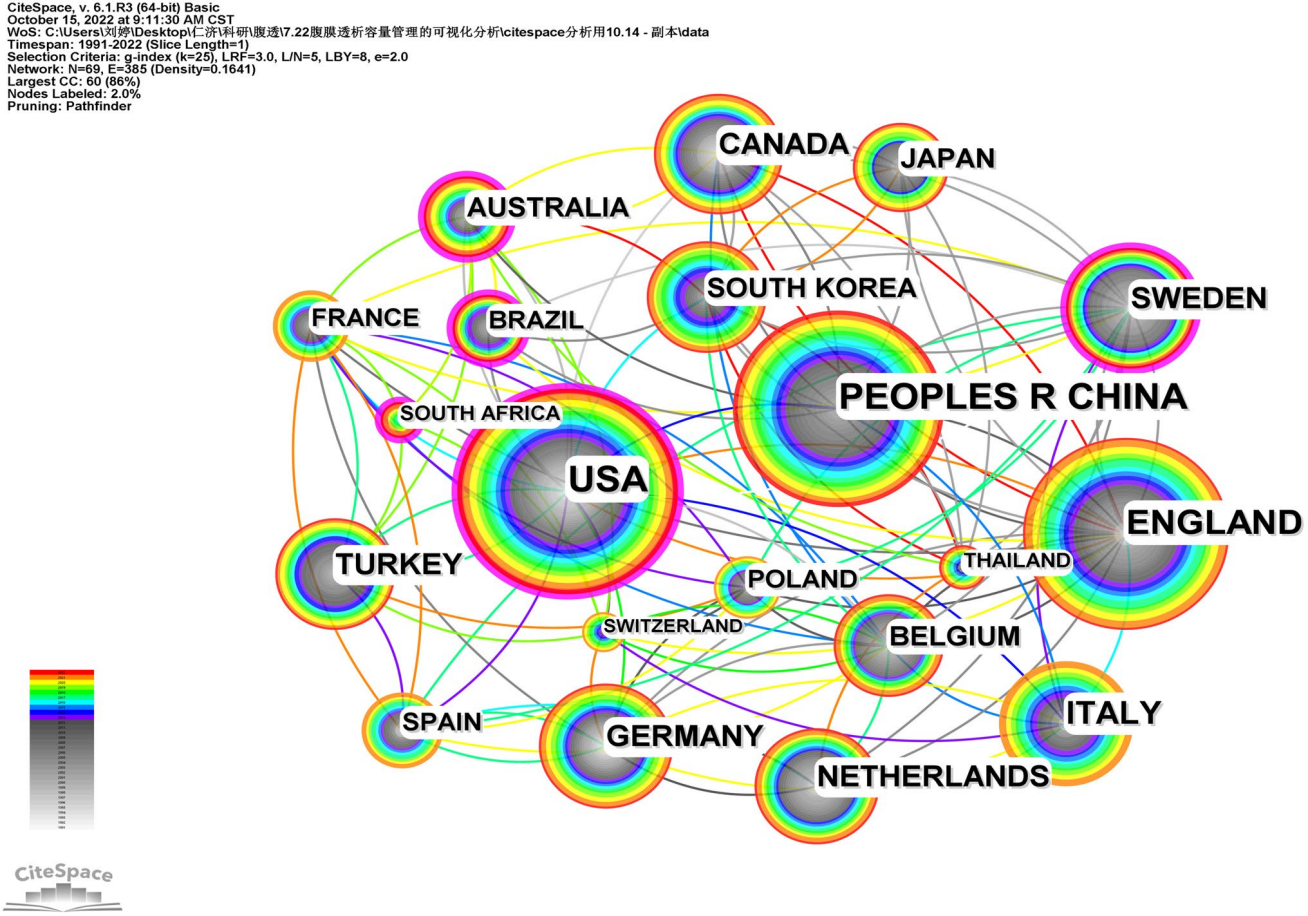
National collaboration network

The top three countries with the highest publication volume in WOS are American (132 articles), China (127 articles), and Britain (118 articles), indicating that China, , and Britain are the core research countries for PD volume management (Fig. 2 and Table 1). The top three countries with intermediary centrality are Australia (0.18), American (0.15), and Sweden (0.13), indicating that these countries play an important leading role in current research on PD volume management and have a deep influence in this field. China, Turkey, and South Korea only published their first literature in this field after the twenty-first century. Although they started late, the total number of articles is high. China’s development speed is fast, and the annual average number of articles is the first.

**Table 1:** Top ten countries in the literatures on PD volume management research

Number	Country	Total number of publications	Intermediary centrality	First article year
1	American	132	0.15	1991
2	China	127	0.02	2001
3	Britain	118	0.09	1992
4	Italy	69	0.03	1997
5	Germany	60	0.02	1992
6	Canada	54	0.03	1991
7	Netherlands	51	0.04	1994
8	Sweden	50	0.13	1992
9	Turkey	48	0.04	2001
10	South Korea	45	0.04	2002

### Research institutions’ distribution

**Fig. 3:**
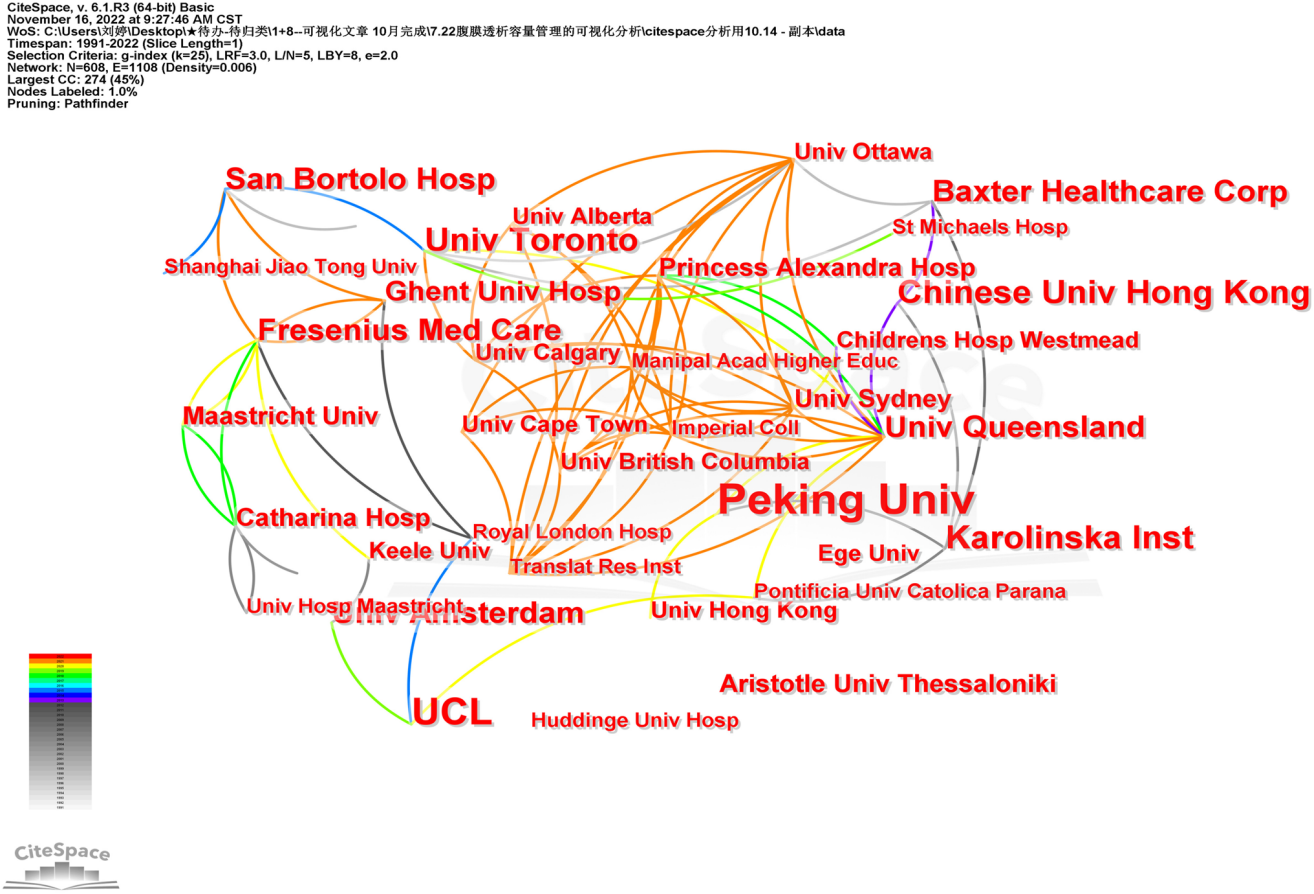
Institution collaboration network

The publishing institutions of PD volume management literatures indexed by WOS are mostly concentrated in higher education institutions, and medical companies and hospitals have also published multiple related literatures. The institutions that have published more articles are Peking Univ (43 articles), UCL (29 articles), and Karolinska Inst (19 articles). Among medical companies, Baxter Healthcare Corp (15 articles) and Fresenius Med Care (14 articles) hold a leading role in this research field (Fig. 3 and Table 2).

**Table 2:** Top ten institutions in the literatures on PD volume management research

Number	Institution	Country	Total number of publications	First article year
1	Peking Univ	China	43	2005
2	UCL	Britain	29	2009
3	Karolinska Inst	Sweden	19	2000
4	Chinese Univ Hong Kong	China	18	2001
5	Univ Toronto	Canada	17	1998
6	Baxter Healthcare Corp	American	15	1998
7	Univ Queensland	Australia	15	2005
8	San Bortolo Hosp	Italy	14	1998
9	Fresenius Med Care	Germany	14	1999
10	Univ Amsterdam	Netherlands	13	1999

### Author distribution

**Fig. 4:**
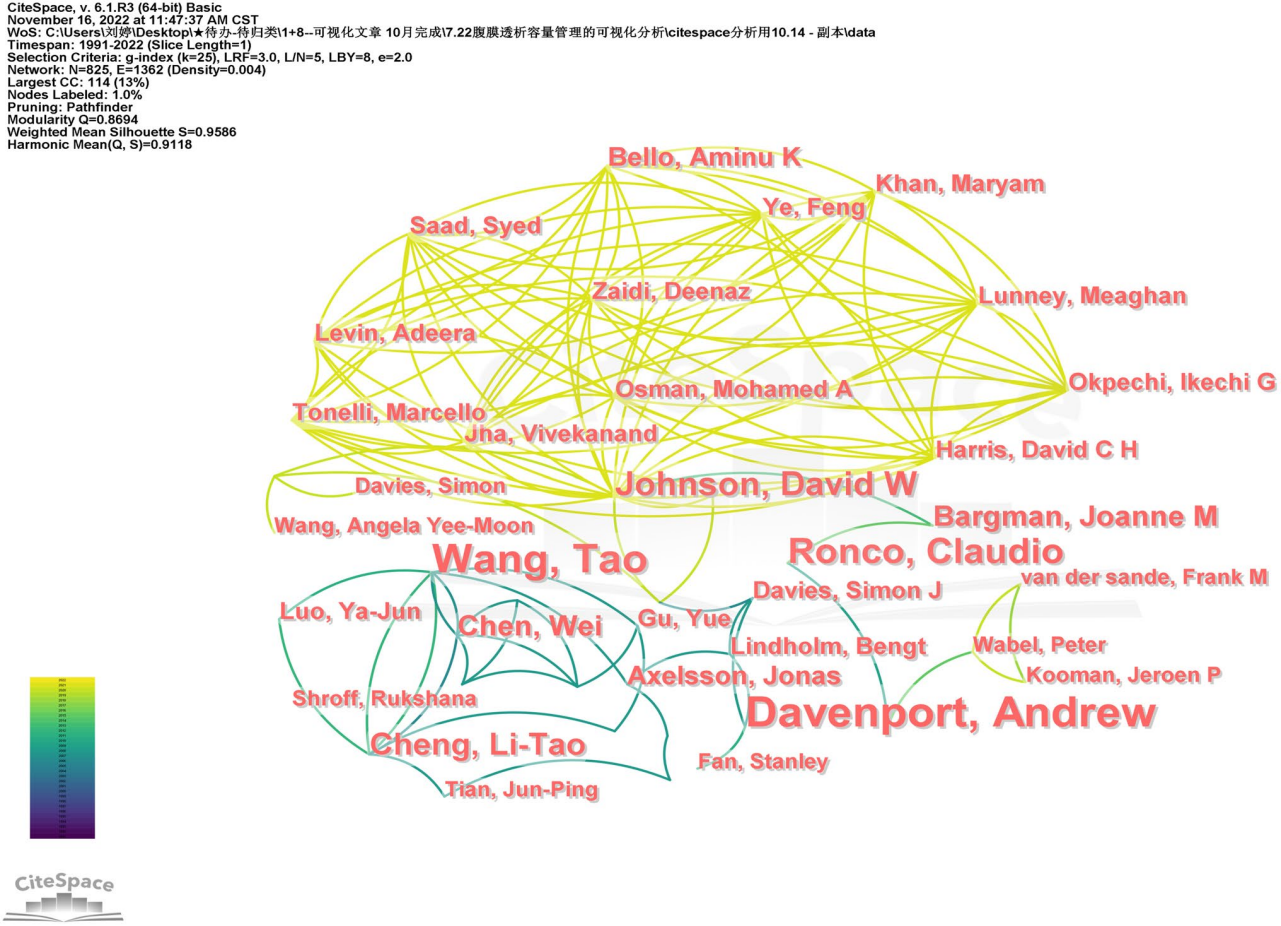
Author collaboration network

From the author collaboration network in Fig. 4 (with a publication volume of ≥ 3), it can be seen that the connections between authors are relatively close. The author collaboration network with Tao Wang (29 articles) as the core has the highest number of publications, with a concentration of publications from 2007 to 2012. The second place is Andrew Davenport (27 articles), with a focus on publications from 2015 to 2021. In recent years, their research contributions in the field of PD patient volume management have been prominent.

### Keyword analysis

Keywords are a highly condensed and summarized content of literature content. Analyzing keywords can obtain the core content of the literatures, help to understand and grasp the research hotspots in the discipline, and predict future development trends. This study merged similar keywords before analysis, such as merging fluid into volume.

### Co-occurrence analysis

**Fig. 5:**
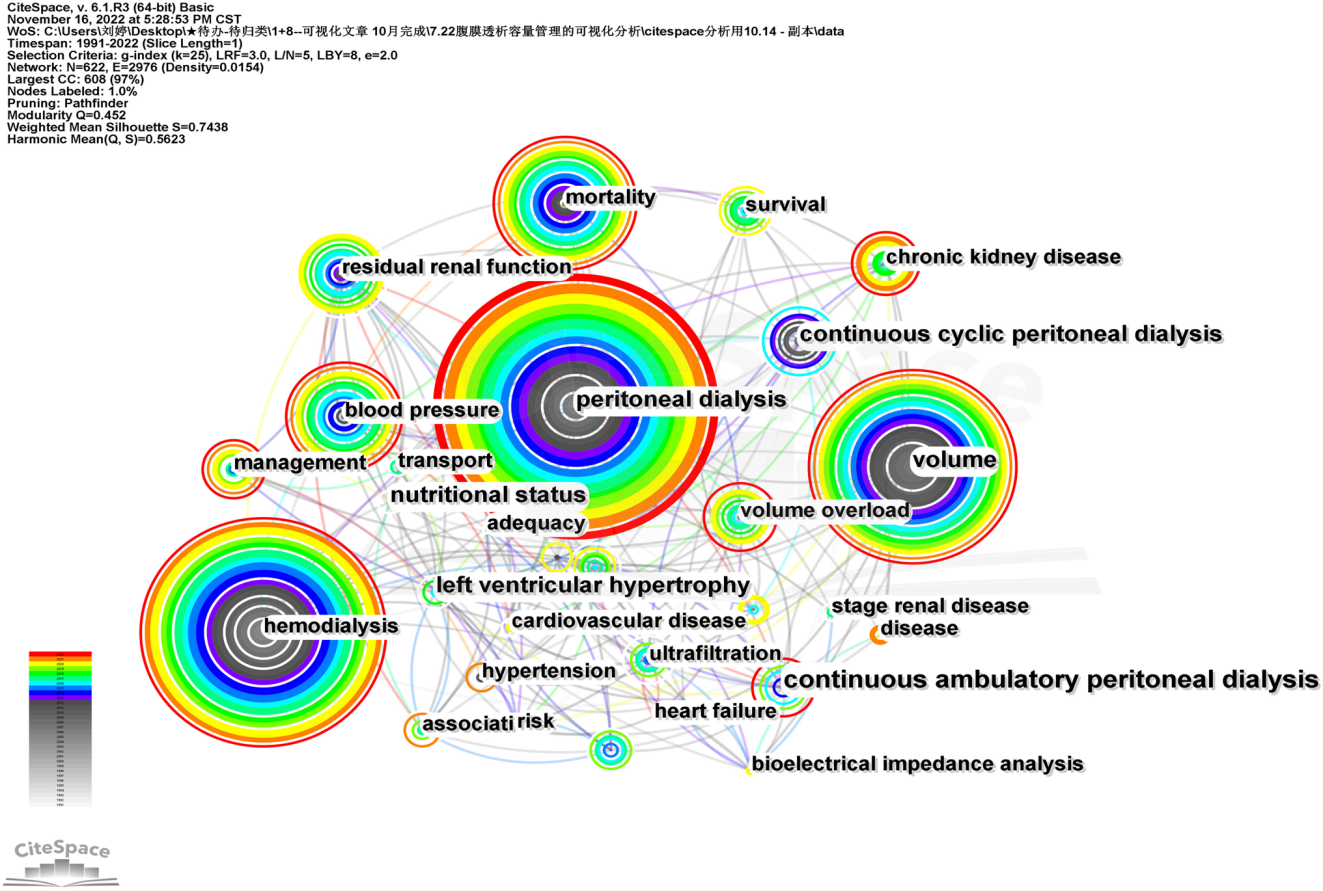
Keyword co-occurrence graph

Keyword node was selected to perform keyword co-occurrence visualization analysis on the literatures (Fig. 5). As the most fundamental terms in the field of PD volume management, the keywords ‘periodic dialysis’ and ‘volume’ have appeared 367 and 251 times, respectively. The topic keywords related to the search term were removed, and the top 20 keywords in terms of frequency are shown in Table 3. The top 3 high-frequency keywords in the ranking are mortality (163 times), blood pressure (137 times), and residual renal function (120 times).

**Table 3:** Top 20 keywords

Number	Keyword	Frequency	Number	Keyword	Frequency
1	Mortality	163	11	Nutritional status	56
2	Blood pressure	137	12	Inflammation	55
3	Residual renal function	120	13	Stage renal disease	48
4	Continuous cyclic peritoneal dialysis	95	14	Cardiovascular disease	48
5	Chronic kidney disease	87	15	Icodextrin	46
6	Survival	86	16	Hypertension	44
7	Continuous ambulatory peritoneal dialysis	85	17	Transport	43
8	Ultrafiltration	65	18	Adequacy	41
9	Body composition	64	19	Risk	39
10	Left ventricular hypertrophy	61	20	Bioimpedance spectroscopy	38

### Cluster analysis

**Fig. 6:**
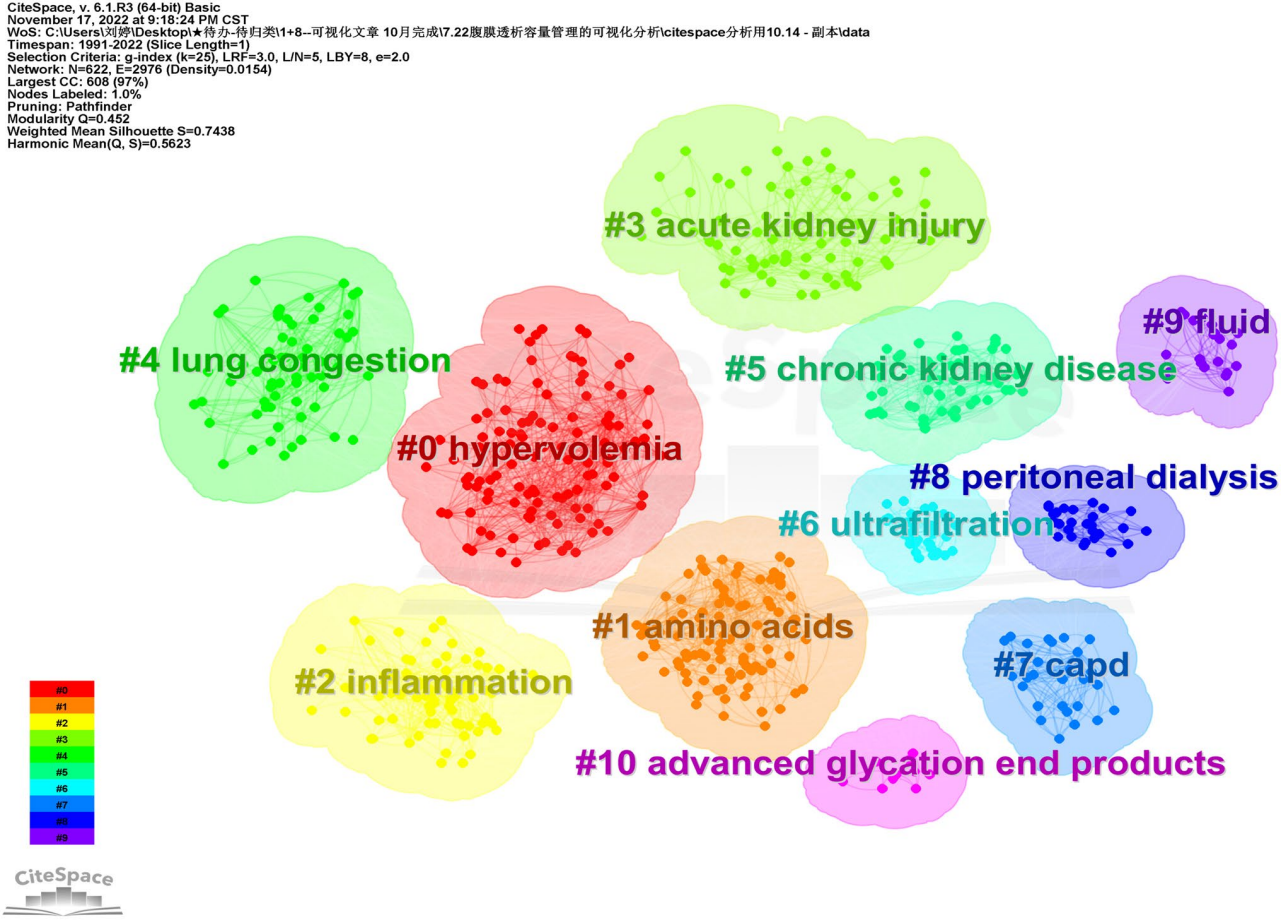
Keyword clustering graph

On the basis of keyword co-occurrence analysis, the LLR algorithm was used for clustering analysis to form a total of 11 cluster groups from # 0 to # 10. The cluster label number increased as the cluster size decreased, and the visualization results are shown in Fig. 6. The modular value of keywords clustering *Q* = 0.452 > 0.3 and the average contour value of clustering *S* = 0.7438 > 0.5 indicate that the clustering structure is significant and has high reliability. Each cluster represents a research direction with high homogeneity, which can represent the leading research topics in the field of PD patient volume management.

### Emergence analysis

**Fig. 7:**
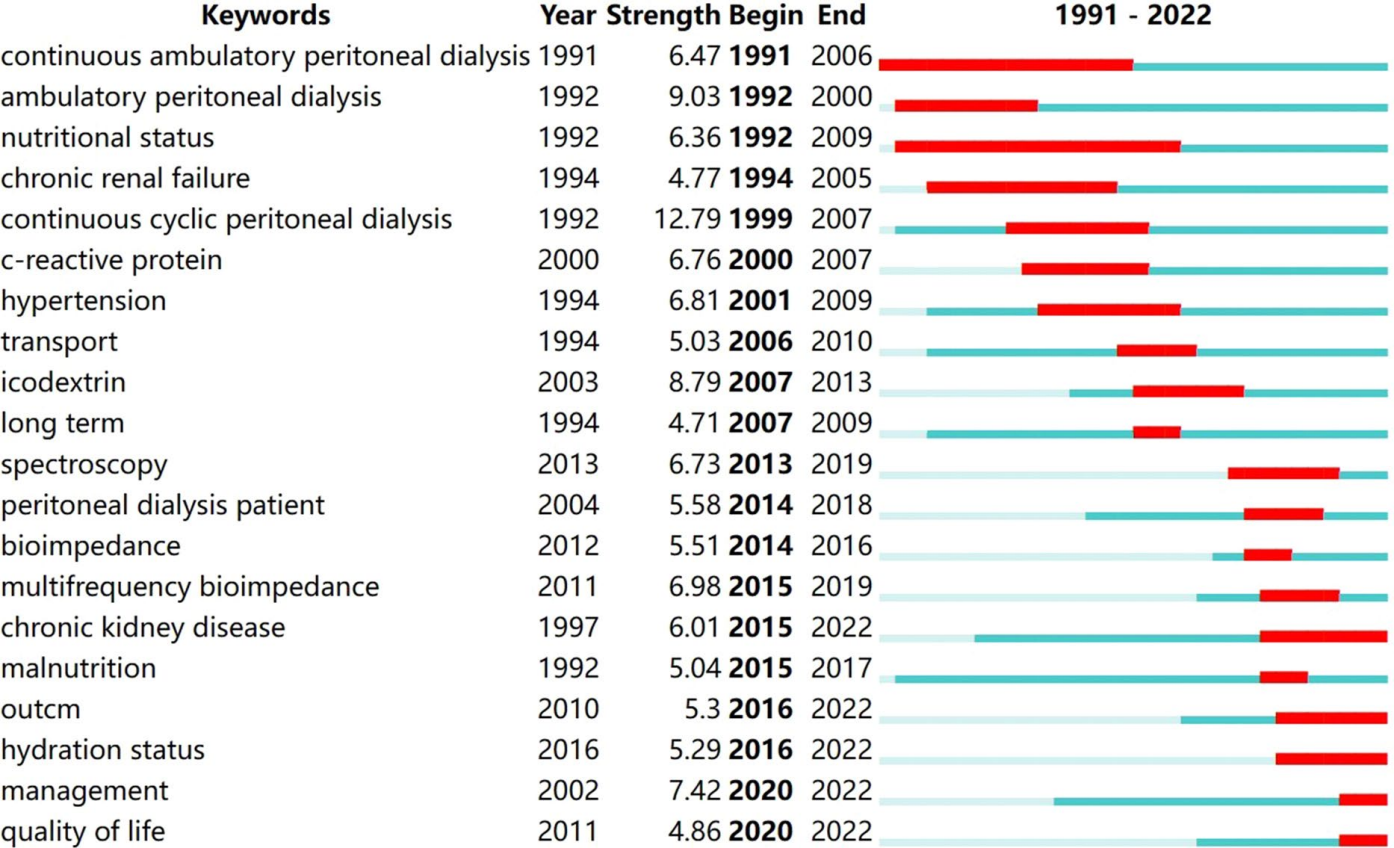
Top 20 Keywords with the strongest citation bursts

The top three keywords in terms of burst intensity are continuous cyclic peritoneal dialysis, ambulatory peritoneal dialysis, and icodextrin (Fig. 7). From 1991 to 2001, the main keywords were continuous cyclic peritoneal dialysis, ambulatory peritoneal dialysis, and nutritional status. From 2002 to 2012, the main keywords were hypertension, transport, and long term. From 2013 to 2022, the main keywords were bioimpedance, management, and quality of life.

## Discussion

Based on the WOS core collection database, this study applied CiteSpace software to conduct a literature metrology analysis of the research on the volume management of PD patients. A total of 788 related literatures were included, and the annual publication volume fluctuated. The country with the highest number of publications is the United States, while China ranks second, indicating that PD volume management is highly valued domestically and has received high attention in this field. However, the intermediary centrality value of scientific research cooperation between China and other countries is relatively low, indicating that there is less cooperation between China and other countries. In the future, it is necessary to strengthen international academic exchanges and cooperation in the field of PD patient volume management. In addition, two medical companies from the top ten institutions are listed, indicating that relevant medical companies in the field attach great importance to volume management for PD patients and promote academic progress by conducting relevant research. Keyword co-occurrence analysis shows that keywords with a higher frequency of occurrence are mostly focused on volume status assessment, dialysis mode, patient outcomes, indicating that volume assessment methods are still a current research hotspot, and more attention is paid to the impact of using different dialysis modes on patient volume status. Cluster analysis forms 11 cluster labels, with each cluster representing research directions that represent cutting-edge topics in the field of PD patient volume management. The emergence analysis shows that the top three keywords in the emergence intensity ranking are continuous circulating peritoneal dialysis, ambulatory peritoneal dialysis, and icodextrin, indicating that researchers should focus on PD mode and peritoneal dialysis fluid selection in the volume management process of PD patients, and dynamically adjust PD plans based on patient conditions. With the change of years and the overall environment, researchers have shifted their focus from the selection of simple PD models to the evaluation and analysis of patient volume status, and now gradually focus on volume management and quality of life.

The high-frequency keywords related to the research hotspots include body composition, blood pressure, etc. Volume overload is closely related to the complications and prognosis of PD, and accurate assessment of patients' volume status is particularly important. A multi-center study [7] evaluated the volume status of patients using a body composition monitor (BCM), and defined the ratio of excessive hydration (OH) to extracellular water (ECW) OH/ECW > 15% as volume overload based on BCM measurement results. The volume status of patients undergoing continuous ambulatory peritoneal dialysis (CAPD) can be simply represented by the ratio of bioimpedance (RBI), which is the impedance at 50 kHz/the impedance at 500 kHz [8]. When BCM analysis cannot be used, RBI can be used as an alternative. Many randomized controlled studies [9–11] assessed the volume status of PD patients through bioelectrical impedance analysis (BIA), and guided volume management. Skin fold thickness and BIA are effective methods in clinical practice when evaluating the volume status of PD patients [12]. Changes in BMI in PD patients should be considered as a result of weight changes caused by volume overload [13]. Edema, lung rales, and weight gain are all clinical signs of volume overload. In addition, the blood pressure can also reflect the patient’s volume status. The study by Park et al. [14] suggests that elevated plasma concentrations of N-terminal B-type natriuretic peptide precursor (NT-proBNP) and adrenomedullin can be used to predict volume overload. For hypertensive patients receiving PD treatment, serum B-type natriuretic peptide (BNP) should be tested to evaluate volume status [15]. In existing technologies, echocardiographic measurements of inferior vena cava diameter and natriuretic peptides (such as BNP and NT-proBNP) can also be used to evaluate volume status [16], but the results are greatly influenced by cardiac status and have not yet been widely used in the PD population.

This study extracted high-frequency keywords related to this research hotspots, including continuous cyclic peritoneal dialysis, continuous ambulatory peritoneal dialysis, icodextrin, etc. The application of automated peritoneal dialysis (APD) during emergency initiation of PD is feasible, and the combination of APD and CAPD is more beneficial for patients without increasing the incidence of early complications [17]. Xie [18] compared the safety of tidal peritoneal dialysis (TPD) and intermittent peritoneal dialysis (IPD) modes in end-stage kidney disease patients after emergency initiation of APD. Both groups of patients underwent 14 days of low volume APD–IPD/TPD treatment before switching to CAPD treatment mode and were followed-up for 2 years. The results showed that the incidence of catheter-related complications in the TPD group was significantly lower than that in the IPD group. APD is the most cost-effective and has the lowest impact on patients’ quality of life among the Mexican population, making it the preferred dialysis method [19].

The traditional PD penetrating agent is glucose. In recent years, icodextrin has been introduced into clinical treatment. Icodextrin helps with volume management in PD patients. Compared with glucose-based peritoneal dialysis, icodextrin has stronger ultrafiltration ability and can improve peritoneal ultrafiltration during long-term abdominal retention, reducing the incidence of volume overload [20]. For patients with good residual kidney function, a single daily use of icodextrin can form effective ultrafiltration and achieve sufficient solute clearance rate. For patients with ultrafiltration failure, use icodextrin twice a day to achieve more ultrafiltration and reduce peritoneal damage caused by glucose exposure. The ultrafiltration ability and sodium ion clearance rate of the mixed dialysate of 7.5% icodextrin and 3.86%/1.36% glucose dialysate are superior to those of icodextrin or glucose dialysate alone [21]. Ö berg [22], based on the three-pore model, explored that APD can significantly improve glucose absorption and achieve sufficient water and solute clearance rates when using a bimodal treatment regimen (i.e., dehydration with high glucose concentration and toxin clearance with low glucose concentration). In clinical practice, patients should be comprehensively evaluated, selecting dialysis modes and dialysates that are suitable for PD patients, and dynamically and flexibly adjusting dialysis plans based on the evaluation results of volume status. In addition, managing volume in peritoneal dialysis requires an individualized approach that combines multiple clinical, dialysis treatment, and patient factors [23].

This study extracted high-frequency keywords related to this research hotspot, including mortality, survival, left ventricular hypertrophy, nutritional status, inflammation, cardiovascular disease, hypertension, risk, etc. Volume overload is common in PD patients and is associated with hypertension, left ventricular hypertrophy, and dysfunction, which are important predictive indicators of mortality in dialysis patients. The prevalence of refractory hypertension in PD patients is 30%, with 38% of patients in a state of volume overload, indicating that volume management can improve the hypertension situation of PD patients [24]. Left ventricular hypertrophy is a risk factor for cardiovascular death in patients with PD. In patients with high peritoneal permeability, icodextrin can be used to improve left ventricular hypertrophy by reducing volume overload [25, 26]. Peritoneal protein loss is a potential issue in PD patients, associated with high mortality, malnutrition, inflammation, and cardiovascular events [27–29]. Excessive hydration is a strong predictor of peritoneal protein loss, emphasizing the importance of volume overload control in PD patients [30]. It is recommended to adjust dialysis schedules and limit water intake for volume management. Clinical nursing staff should not only pay attention to various physiological indicators of PD patients, but also strengthen health education for patients, increase their attention to health outcomes, cooperate with doctors, nurses, and patients, and achieve the common goal of reducing the incidence of complications.

Compared with the traditional literatures review, the visual analysis of literatures metrology is more intuitive and comprehensive, but this study also has some limitations. First, CiteSpace can only analyze one database. In this study, only the WOS core collection database was searched, and other large medical databases such as PubMed, Scopus, Embase, etc., were not searched. Language and literature types were restricted, and some studies may be ignored when manually removing irrelevant literatures. However, WOS is the most commonly used database in literatures metrology research. To some extent, this study can still explain the research focus and development trend of PD patient volume management. Another drawback is that the data analyzed in this study rely on database statistics and cannot provide in-depth analysis of the article content.

## Conclusion

This study applies CiteSpace to the field of PD patient volume management, and reviews and analyzes relevant literatures. The research on PD volume management in China started late, but has developed rapidly. The research results of domestic higher education institutions are abundant, and they firmly grasp the current research hotspots. The research content is at the forefront, but there is little cooperation with other countries. Therefore, it is necessary to strengthen international communication and cooperation, conduct multi-center research, and explore research ideas and build broader research platforms to obtain more comprehensive data. This study uses the research method of literatures metrology visual analysis atlas to present current research hotspots and trends, and draws the conclusion that the research hotspots of volume management for PD patients are in the following three aspects: volume status assessment, dialysis scheme selection and adjustment, and attention to health outcomes. It is recommended that clinical medical staff pay more attention to the management of PD patient volume, extensively explore and utilize big data information, and combine it with clinical practice to better carry out research on PD patient volume management.

## Data Availability

The data generated in this study has been included in the article, and further inquiries can be directed to the corresponding author or the frst author.
